# Five-Year Cardiorenal Outcomes and Longitudinal Chronic Kidney Disease Screening in People with Type 2 Diabetes Managed by Endocrinologists: A Nationwide Retrospective Cohort Study

**DOI:** 10.3390/medsci14040506

**Published:** 2026-08-21

**Authors:** José Ignacio Martínez-Montoro, José Juan Aparicio-Sánchez, Belén Pimentel, Mónica Juárez-Campo, Maria Luisa Alamillo, Yesika Díaz, José Carlos Fernández-García

**Affiliations:** 1 Department of Endocrinology and Nutrition, Hospital Universitario Virgen de la Victoria, IBIMA Plataforma BIONAND, Instituto de Investigación Biomédica de Málaga, Faculty of Medicine, University of Málaga, 29010 Málaga, Spain; 2 Centro de Investigación Biomédica en Red de la Fisiopatología de la Obesidad y la Nutrición (CIBERObn), Instituto de Salud Carlos III, 28029 Madrid, Spain; 3 Medical Department, BioPharmaceuticals, AstraZeneca, 28046 Madrid, Spain; josejuan.aparicio@astrazeneca.com (J.J.A.-S.); belen.pimentel1@astrazeneca.com (B.P.); monica.juarez@astrazeneca.com (M.J.-C.); 4 Telómera SLU, 28001 Madrid, Spain; marisa.alamillo@telomera.com (M.L.A.); yesika.diaz@telomera.com (Y.D.); 5 Department of Endocrinology and Nutrition, Hospital Regional Universitario de Málaga, IBIMA Plataforma BIONAND, Instituto de Investigación Biomédica de Málaga, Faculty of Medicine, University of Málaga, 29010 Málaga, Spain; 6 Centro de Investigación Biomédica en Red de Diabetes y Enfermedades Metabólicas Asociadas (CIBERDEM), Instituto de Salud Carlos III, 28029 Madrid, Spain

**Keywords:** chronic kidney disease screening, endocrinology care, risk stratification, type 2 diabetes, UACR screening, cardiorenal outcomes, albuminuria, eGFR monitoring

## Abstract

Background/Objectives: Type 2 diabetes (T2D) is a major risk factor for cardiovascular disease and chronic kidney disease (CKD), yet the extent of longitudinal CKD screening in routine clinical practice remains unclear. This study aimed to assess long-term cardiorenal outcomes and CKD screening practices in patients with T2D managed across primary care and endocrinology settings, evaluating the feasibility of risk stratification in real-world practice. Methods: We conducted a retrospective cohort study using anonymized data from the Telotrón database (2.2 million patients). Adults with T2D attended across primary and endocrinology care were followed from 1 January 2018 to 31 December 2022. Frequency of estimated glomerular filtration rate (eGFR) and urine albumin-to-creatinine ratio (UACR) measurements, and cumulative proportion of kidney and cardiovascular events, hospitalizations, and mortality were assessed. Results: Among 13,600 individuals, 31.1% experienced a kidney event and 18.6% a cardiovascular event over 5 years. All-cause hospitalization occurred in 24.9% and 16.1% died. First occurrence of an abnormal eGFR or UACR measurement occurred in 37.0% of participants without baseline CKD, and 45.4% with baseline CKD showed progression. Annual monitoring was limited, with 39.1% and 16.6% undergoing at least one yearly assessment, and 2.9 and 1.5 mean number of measurements per patient for eGFR and UACR, respectively. Conclusions: Despite receiving endocrinology care, kidney disease progression and cardiovascular events remain a major concern in T2D, and yet suboptimal albuminuria and eGFR assessment limit early detection and risk stratification, thereby hindering risk-directed management.

## 1. Introduction

Type 2 diabetes (T2D) is a global public health challenge, affecting more than 500 million adults aged 20–79 years. T2D is a major risk factor for cardiovascular disease (CVD), heart failure (HF), arrhythmias, and chronic kidney disease (CKD) [1,2,3]. CKD is frequently observed in T2D, with a prevalence of approximately 40%, representing a crucial mediator of elevated CVD risk in this population [4,5,6,7].

Post-hoc and subgroup analyses of large-scale trials, such as ACCORD and ADVANCE [8,9], have reported substantially higher CVD event risk among people with T2D and CKD compared with those with T2D alone. In addition, estimated glomerular filtration rate (eGFR) slope decline has been associated with increased risk of major kidney and macrovascular events and all-cause mortality, and CKD severity (as examined in FIELD [10]) has been described as a determinant of CVD risk in T2D. Albuminuria has also been identified as an independent risk factor for atherosclerotic CVD (ASCVD) and HF in patients with T2D and CKD [11].

Risk prediction equations were developed to estimate the 5-year probability of incident reduced kidney function (eGFR < 60 mL/min/1.73 m^2^), with models developed separately for individuals with diabetes and incorporating routinely available variables including eGFR and albuminuria; these models were positioned as potentially implementable within electronic health records [12]. Albuminuria in early-stage CKD has been independently associated with higher risks of MACE, HF, and all-cause mortality, and significantly modified the relationship between CKD stage and outcomes [13].

Although T2D care often begins in primary care, a substantial proportion of patients are managed in endocrinology clinics, where clinical complexity and comorbidity burden are typically higher [6,14,15].

In the ENDO-CKD study, we previously reported a 40.7% CKD prevalence in a nationwide cohort of patients with T2D managed across primary and endocrinology care settings [16]. Here, we report a 5-year longitudinal analysis describing cardiorenal outcomes, hospitalizations, and mortality alongside longitudinal eGFR and UACR testing patterns to contextualize event burden within real-world kidney biomarker ascertainment.

## 2. Materials and Methods

### 2.1. Study Design and Population

This is a retrospective observational study with data collected from the Telotrón^®^ database (Telómera SLU, Madrid, Spain), which contains anonymized records from the Spanish National Health System, covering both primary and hospital care settings across seven Autonomous Communities in Spain, encompassing a population of over 2.2 million individuals. The database has been validated as a reliable source for real-world evidence research in Spain [17,18].

The study design has been previously described [16]. Here, we present the longitudinal analysis of individuals meeting inclusion criteria on 1 January 2018 (index date) who were followed until lost to follow-up, death, or the end of the observation period (31 December 2022). This cohort includes adults aged 18 years or older with a type 2 diabetes diagnosis and at least one recorded visit to an Endocrinology service on or after the time of type 2 diabetes diagnosis. Individuals were required to have available follow-up information after the index date. Type 2 diabetes was defined based on the presence of an ICD diagnosis code, a glycated hemoglobin (HbA1c) value ≥6.5%, or a prescription for any glucose-lowering medication. Individuals with type 1 diabetes or gestational diabetes were excluded. The complete inclusion and exclusion criteria are detailed in Table S1 and International Classification of Diseases (ICD) codes and Anatomical Therapeutic Chemical classification codes (ATC) in Tables S2 and S3 respectively.

The study was conducted in accordance with the Declaration of Helsinki and approved by the Ethics Committee of San Carlos Clinic Hospital (Madrid, Spain), approval code 23/653-E (31 October 2023).

### 2.2. Study Variables and Outcomes

#### 2.2.1. CKD Definition

CKD was defined as the presence of a diagnosis code or at least one recorded measurement of either eGFR < 60 mL/min/1.73 m^2^ or UACR ≥ 30 mg/g documented on or before the index date (the value closest to the index date was used). When eGFR value was not available, it was estimated using the 2021 CKD-EPI equation [19]. CKD was evaluated both as a comorbidity at baseline and as an outcome during the 5-year observation period in the subgroup without CKD at baseline. For baseline CKD assessment, we used the closest available eGFR or urine albumin-to-creatinine ratio (UACR) value to the index date, regardless of whether it was within or outside the normal range. During follow-up, development of CKD was defined as the first pathological value of eGFR or UACR.

A single pathological value of either eGFR or UACR was considered to define CKD, rather than requiring two abnormal measurements at least three months apart, as per KDIGO recommendations [20]. This approach has been deemed the most appropriate for the study’s objective, as discussed in the previous manuscript and other studies [16,21,22,23,24]. CKD definition as per KDIGO recommendation could potentially lead to underestimation of CKD prevalence (especially when using data from EHRs) and would not reflect routine clinical practice. In accordance with the prespecified data extraction protocol, only the first qualifying abnormal eGFR or UACR measurement after the index date was extracted; subsequent measurements were therefore not available to assess persistence of the abnormality. In addition, we considered that this CKD-definition criteria would allow identification of most potential patients with CKD.

#### 2.2.2. Demographic, Clinical Variables, and Medication Use

Baseline demographic and clinical variables included age, sex, body mass index (BMI), systolic and diastolic blood pressure, key biochemical indicators (eGFR, UACR, HbA1c, serum potassium, high-density lipoprotein (HDL) cholesterol, low-density lipoprotein (LDL) cholesterol, and triglycerides), and comorbidities (CKD, peripheral artery disease (PAD), stroke, myocardial infarction (MI), angina pectoris, HF, atrial fibrillation/flutter, hypertension, hyperlipidemia, diabetic retinopathy, diabetic neuropathy, obesity, and hyperkalemia). Baseline variables were defined using the most recent value within the two years prior to the index date (missing if unavailable).

Chronic medication use was defined by ≥1 prescription within the six months prior to index [25], for any of the following drug classes: renin–angiotensin–aldosterone system inhibitors (RAASi), beta blockers, calcium channel blockers, diuretics, lipid-lowering drugs, anti-platelet agents, glucose-lowering therapies (metformin, sodium-glucose cotransporter-2 inhibitors (SGLT2i), dipeptidyl peptidase-4 inhibitors (DPP4i), glucagon-like peptide-1 receptor agonists (GLP-1 RAs), sulfonylureas, glinides, and insulins), and potassium binders.

The International Classification of Diseases, 9th Edition, Clinical Modification (ICD-9-CM) and 10th Revision, Clinical Modification (ICD-10-CM), and the Anatomical Therapeutic Chemical (ATC) classification codes were used. Complete lists of ICD and ATC codes included in this study have been previously reported [16].

#### 2.2.3. Long-Term Clinical Outcomes

The first occurrence of selected cardiorenal outcomes including the first occurrence of an abnormal eGFR or UACR measurement, kidney events, and cardiovascular events were assessed over the 5-year observation period. Hospitalizations (all-cause and HF-related) and all-cause mortality were also assessed. First occurrence of an abnormal eGFR or UACR measurement was described in the subgroup without CKD at baseline. Cardiovascular events included HF, MI, PAD, and stroke. Kidney events were defined as worsening of kidney function during follow-up by a decline in eGFR > 30% or worsening of UACR category (based on KDIGO classification), or the development of end-stage kidney disease (ESKD), defined as sustained eGFR < 15 mL/min/1.73 m^2^, initiation of dialysis, or kidney transplantation. Analyses were conducted for the overall study population and stratified by baseline CKD presence.

#### 2.2.4. CKD Screening

CKD screening over time was assessed using two complementary approaches evaluating measurements of eGFR and UACR separately during the 5-year observation period. First, by calculating the average number of measurements per patient during the period. Second, estimating the number and proportion of individuals with at least one annual measurement. The total baseline study population was used as the denominator in both approaches to capture overall screening coverage across the complete population. The average number of visits to the endocrinologist per patient during the observation period was also evaluated.

### 2.3. Statistical Analysis

#### 2.3.1. Demographic, Clinical Characteristics, Comorbidities, and Medication Use

Demographic and clinical characteristics, comorbidities, and medication use were described using the most recent data available closest to the index date. Medication use was expressed as the proportion of individuals with at least one prescription for each selected drug class within the six months before index date. Results are presented for the overall study population and for subgroups according to the presence or absence of CKD at baseline.

Categorical variables were reported as counts and percentages, and continuous variables as means with standard deviations (SD) or medians with interquartile ranges (IQR), as appropriate. Unadjusted descriptive summaries were provided for subgroups of interest, along with clinical interpretation of the observed findings. The study adhered to the STROBE and the New England Journal of Medicine Statistical Reporting Guidelines [26,27].

#### 2.3.2. Cumulative Proportion of Clinical Outcomes

The cumulative proportion of cardiorenal events, hospitalizations (all-cause and HF-related), and all-cause mortality was calculated as the crude proportion of individuals experiencing a first recorded event over the 5-year observation period (i.e., number of individuals experiencing the event divided by the total number of individuals in the cohort at baseline, regardless of follow-up duration). Only the first occurrence of each event type per individual was considered. For kidney-related outcome, only the first occurrence of an abnormal eGFR or UACR measurement was considered. Patients who left the database or reached the administrative end of the study before 5 years were not excluded from the denominator; therefore, these estimates represent observed crude proportions rather than actuarial risk estimates adjusted for censoring or competing events. A sensitivity analysis was conducted to evaluate the impact of the study’s CKD definition in the subgroup of patients who had at least one measurement of both eGFR and UACR at or before the index date.

#### 2.3.3. Multivariate Analysis

A multivariable logistic model was developed to identify baseline risk factors associated with the first occurrence of an abnormal eGFR or UACR measurement. The model was conducted in the subgroup of individuals who had at least one measurement of both eGFR and UACR at index date and were CKD-free at baseline (*n* = 5570). The dependent variable was the presence of CKD during the follow-up. Candidate covariates were prespecified a priori based on clinical relevance, published evidence, data availability, and avoidance of collinearity. Covariates included were age, sex, BMI, HbA1c, eGFR, UACR, systolic and diastolic blood pressure values closest to index date, number of cardiovascular comorbidities, use of lipid-lowering therapy, insulin treatment (considered a proxy for longer diabetes duration, as this data was not available), and number of non-insulin glucose-lowering agents. Univariate analyses were performed, but all prespecified candidate covariates were entered into the initial multivariable model regardless of their statistical significance in the univariate analyses. Statistical significance was set at *p* < 0.05. All analyses were conducted using R (version 4.3.2).

## 3. Results

### 3.1. Baseline Characteristics of the Study Population

As of 1 January 2018, a total of 883,736 adults were active in the Telotrón database. Of these, 70,658 met the inclusion criteria for type 2 diabetes, and 16,480 had at least one recorded visit to an Endocrinology service on or after their type 2 diabetes diagnosis. After excluding individuals with missing or inconsistent data, or without continuous registration in the database, the final study population comprised 13,600 individuals (Figure S1).

The mean age of the cohort was 64.7 years (SD 12.8), and 49.9% were women. Mean HbA1c was 7.4% (SD 1.6), and mean BMI was 31.8 kg/m^2^ (SD 6.5). Regarding kidney parameters, mean eGFR was 79.8 mL/min/1.73 m^2^ (SD 22.6), and median UACR was 12.7 mg/g (IQR 7.1–36.6). Overall, 9492 individuals (69.8%) had both eGFR and UACR measurements available at or before the index date; 15.8% lacked eGFR values and 29.8% lacked UACR data at baseline.

Hypertension and hyperlipidemia were the most common comorbidities, affecting 57.9% and 56.4% of participants, respectively. More than one-third of individuals had CKD (36.2%) and/or obesity (35.0%). Heart failure (HF), atrial fibrillation (AF), and peripheral artery disease (PAD) were present in 8.9%, 8.5%, and 8.8% of the cohort, respectively.

At baseline, 88.4% of participants received glucose-lowering therapy, including metformin (67.3%), insulin (43.6%), DPP-4i (39.7%), SGLT2i (18.3%), or GLP-1 RAs (9.8%). In addition, 64.5% were treated with RAASi, 28.7% with beta blockers, 21.4% with calcium channel blockers, and 24.3% with diuretics, while 68.8% received lipid-lowering therapy.

Baseline clinical characteristics, laboratory values, and treatments for the overall population and stratified by baseline CKD status are shown in Table 1.

### 3.2. Long-Term Clinical Outcomes

**Figure 1:**
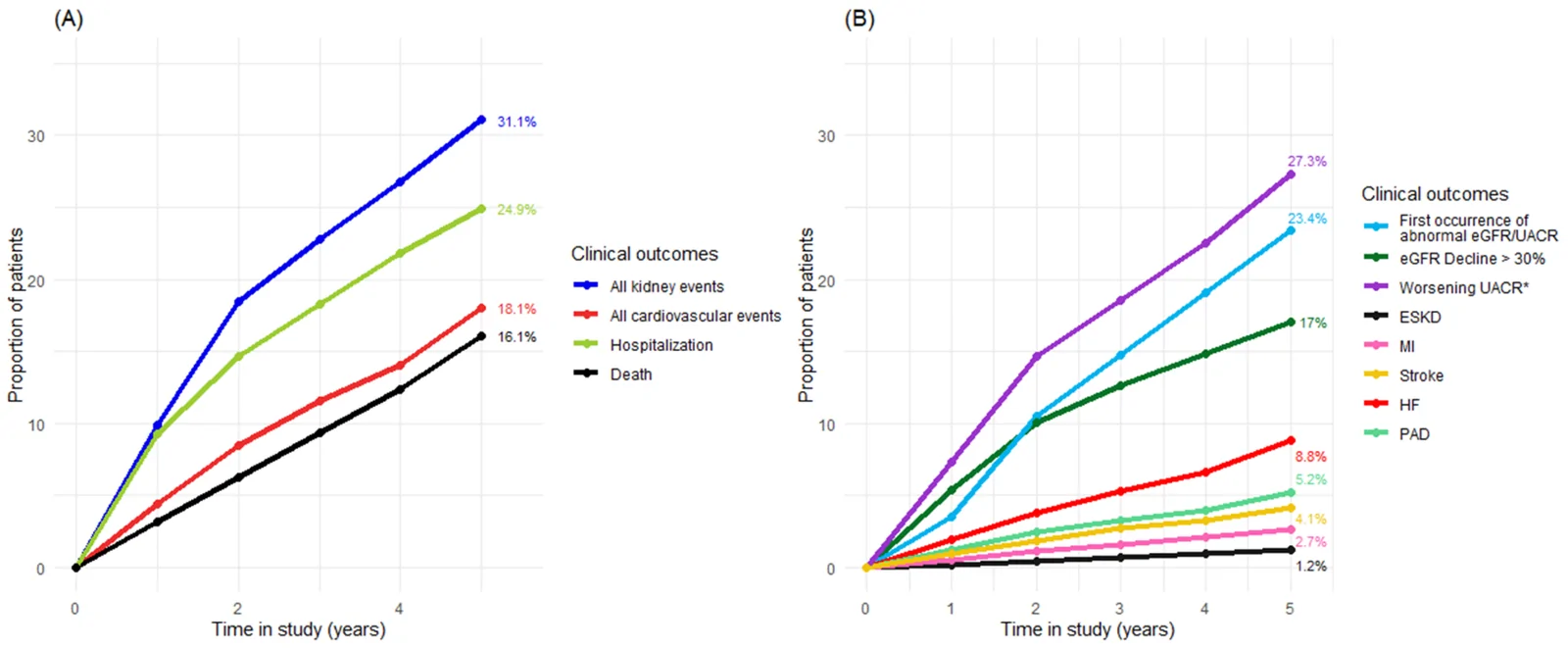
Cumulative proportion of first manifestation of long-term clinical outcomes during a 5-year observation period in the study population. (**A**) Cumulative proportion of first manifestation of all kidney events (blue line), all cardiovascular events (red line), all-cause hospitalizations (green line) and all-cause mortality events (black line). (**B**) Cumulative proportion of the first occurrence of an abnormal eGFR or UACR measurement (blue line), eGFR decline ≥ 30% (dark green line), worsening of UACR (purple line), ESKD (black line), HF (red line), PAD (light green line), MI (pink line), Stroke (yellow line). * Worsening of UACR calculated over the total number of patients with available UACR value.

Over the 5-year follow-up, 31.1% of participants experienced at least one kidney event and 18.6% experienced at least one cardiovascular event. Kidney event results should be interpreted within the context of a broad CKD definition based on a single abnormal value. In the overall cohort, 24.9% had at least one all-cause hospitalization and 16.1% died during follow-up (Figure 1A, Table 2).

When kidney events were examined in detail (Figure 1B, Table 2), 1.2% of patients reached end-stage kidney disease (ESKD), 17.0% experienced a ≥30% decline in eGFR, and 27.3% showed worsening of UACR category. Among cardiovascular outcomes, heart failure (HF) was the most frequent event (8.8%), followed by peripheral artery disease (PAD), stroke, and myocardial infarction (MI), with cumulative proportions of 5.2%, 4.1%, and 2.7%, respectively.

**Figure 2:**
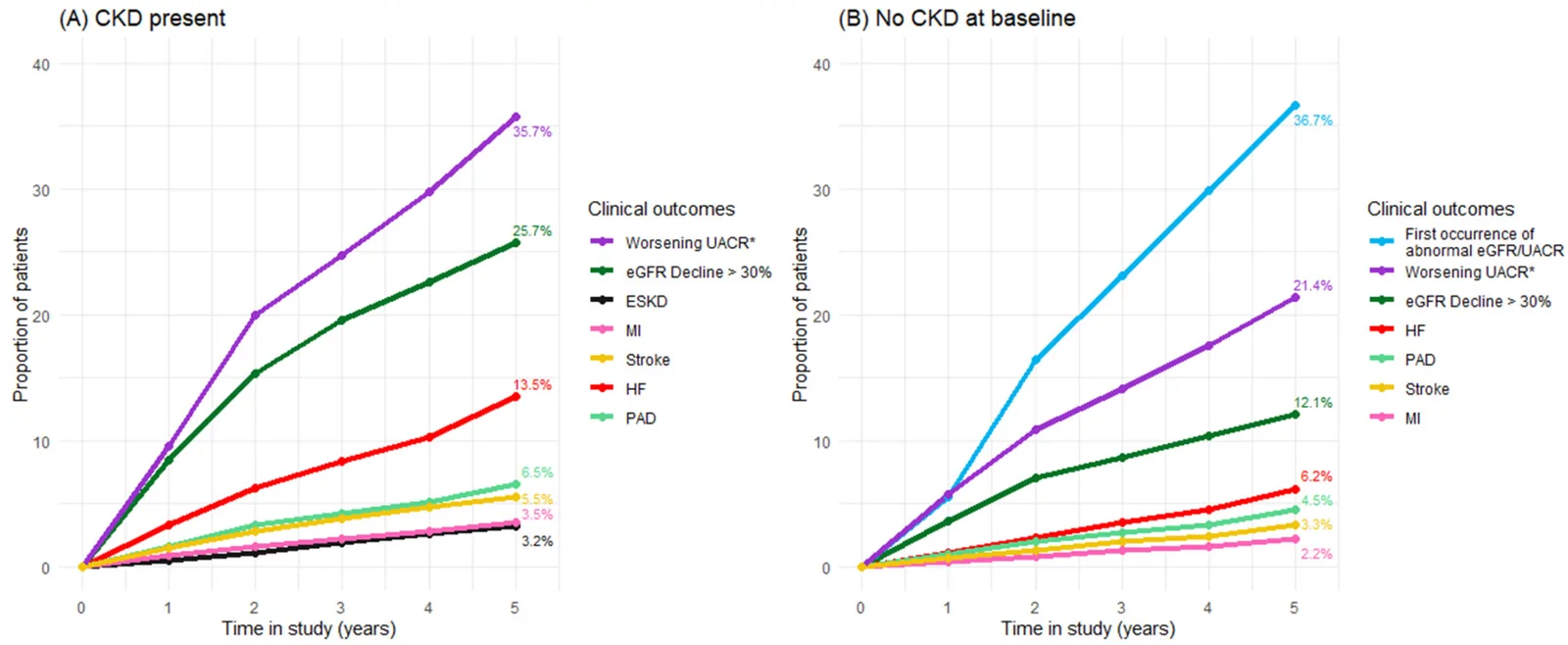
Cumulative proportion of first manifestation of long-term clinical outcomes during a 5-year observation period in the study population by CKD presence (**A**) or absence (**B**) at baseline. It is shown the cumulative proportion of the first occurrence of an abnormal eGFR or UACR measurement (light blue line), eGFR decline ≥ 30% (dark green), worsening of UACR category (purple line), ESKD (black line), HF (red line), PAD (light green line), MI (pink line), Stroke (yellow line). * Worsening of UACR calculated over the total number of patients with available UACR value.

Outcomes were further assessed according to baseline CKD status (Figure 2, Table 2). Among participants with CKD at baseline, 45.4% experienced at least one kidney event during follow-up: 3.2% progressed to ESKD, 25.7% had an eGFR decline of ≥30%, and 35.7% worsened their UACR category (Figure 2A, Table 2). In this subgroup, 25.5% experienced at least one cardiovascular event; HF occurred in 13.5%, and PAD, stroke, and MI occurred in 6.5%, 5.5%, and 3.5% of patients, respectively. The cumulative proportion of all-cause hospitalization was 31.1%, and mortality was 27.8% (Table 2).

Among participants without CKD at baseline, 37.0% had the first occurrence of an abnormal eGFR or UACR measurement during follow-up; 12.1% experienced an eGFR decline of ≥30% and 21.4% showed worsening of UACR category. Assessment of CV events showed that 14.7% of patients had at least one CV event, while all-cause hospitalization was 21.5% and mortality was 9.4% (Figure 2B, Table 2).

### 3.3. Assessment of CKD Screening over a Five-Year Follow-Up

Over the 5-year observation period, patients had a mean of 4.5 endocrinology visits (SD 7.6). On average, 2.9 eGFR measurements (SD 2.4) and 1.5 UACR measurements (SD 1.6) were recorded per patient. Overall, 39.1% of individuals had at least one annual eGFR measurement and 16.6% had at least one annual UACR measurement during follow-up (Table 3).

In the group with CKD at baseline, 32.9% of patients had at least one annual eGFR measurement and 15.7% had at least one annual UACR measurement during the 5-year follow-up period. The average number of measurements per patient within the 5-year period was 2.7 for eGFR (SD 2.5) and 1.4 for UACR (SD 1.7) (Table 3).

On the other hand, among individuals without CKD at baseline, 42.6% had at least one annual eGFR measurement and 17.1% had at least one annual UACR measurement during follow-up. The average number of measurements per patient in the 5-year follow-up period in this subgroup was 3.0 for eGFR (SD 2.3) and 1.5 for UACR (SD 1.6) (Table 3).

### 3.4. Sensitivity Analysis

Sensitivity analyses restricted to participants with both eGFR and UACR available at the index date (*n* = 9492) yielded results consistent with the main analyses (Table S4). In this subgroup, 34.7% experienced at least one kidney event and 17.9% experienced at least one cardiovascular event over 5 years. All-cause hospitalization occurred in 25.4% of participants, and 15.5% died during follow-up (Table S4).

When stratified by baseline CKD status, event rates remained similar to those observed in the primary analysis (Table 1). Among participants with CKD at baseline, 50.6% experienced at least one kidney event during follow-up, including a ≥30% eGFR decline in 28.1% and worsening of UACR category in 34.4%. In this subgroup, 24.9% had at least one cardiovascular event, with heart failure being the most frequent (13.0%). The cumulative proportion of all-cause hospitalization and mortality was 31.5% and 26.1%, respectively (Table S4).

Among participants without CKD at baseline, the first occurrence of an abnormal eGFR or UACR measurement occurred in 26.5% during follow-up, with lower rates of worsening UACR category compared with the main analysis (Table S4). Cardiovascular events (12.9%), hospitalizations (21.1%), and mortality (8.1%) were also lower, and remained consistent with the overall pattern observed in the primary analysis (Table S4).

### 3.5. Assessment of Potential Risk Factors for the First Occurrence of an Abnormal eGFR or UACR Measurement

To identify baseline factors associated with the first occurrence of an abnormal eGFR or UACR measurement over 5 years in the ENDO-CKD population, we fitted a multivariable logistic regression model among participants who were CKD-free at baseline and had both kidney biomarkers available at the index date (*n* = 5570). Baseline characteristics for this subgroup are shown in Table S5.

Several variables were independently associated with a higher likelihood of experiencing a first abnormal eGFR or UACR measurement during follow-up (Table S6). Insulin use was associated with increased risk (OR 1.278; 95% CI 1.083–1.509; *p* = 0.004), as were higher baseline UACR (OR 1.070; 95% CI 1.057–1.084; *p* < 0.001), higher BMI (OR 1.023; 95% CI 1.009–1.036; *p* < 0.001), and older age (OR 1.015; 95% CI 1.005–1.025; *p* = 0.002). In contrast, higher baseline eGFR was associated with a lower risk of the first occurrence of an abnormal eGFR or UACR value (OR 0.940; 95% CI 0.932–0.948; *p* < 0.001). Systolic blood pressure was retained in the model but did not reach statistical significance (OR 1.005; 95% CI 1.000–1.010; *p* = 0.057).

Sex, HbA1c, diastolic blood pressure, number of cardiovascular comorbidities, use of lipid-lowering therapy, and the number of non-insulin glucose-lowering agents were not significantly associated with the first occurrence of an abnormal eGFR or UACR measurement in this model (Table S6).

## 4. Discussion

This real-world study provides a 5-year longitudinal characterization of cardiorenal outcomes, hospitalizations, mortality, and—critically—kidney biomarker ascertainment among adults with T2D managed across primary and endocrinology care in Spain [16]. Over follow-up, we observed a substantial burden of kidney and cardiovascular events, alongside frequent all-cause hospitalizations and death, underscoring the high-risk clinical profile of this specialty-managed population. However, these outcomes occurred in the context of incomplete longitudinal CKD screening, particularly for UACR, highlighting an important gap between the clinical risk burden and the routine capture of the biomarkers required for CKD recognition and risk classification [12,13,20].

A key interpretive contribution of this work is that it couples outcome burden with real-world testing patterns for eGFR and UACR in a nationwide cohort of people with T2D managed across care settings. We found that, over 5 years, patients had relatively few recorded measurements on average, and only a minority had at least one annual assessment, with the shortfall more pronounced for UACR than eGFR. This screening gap matters because albuminuria is not merely a diagnostic adjunct as converging evidence also indicates that albuminuria carries prognostic information beyond eGFR alone, particularly early in CKD [2]. Therefore, incomplete UACR ascertainment may limit the ability to recognize clinically meaningful risk heterogeneity—particularly among individuals who may have preserved eGFR but elevated albuminuria—and may constrain prognostic interpretation of observed cardiovascular outcomes in routine care.

In parallel, multinational CKD-PC analyses demonstrate that 5-year risk prediction for incident reduced eGFR can be derived from routinely available variables and was explicitly developed in diabetes-specific models that incorporate both baseline eGFR and albuminuria, alongside other clinical characteristics, with potential for integration into electronic health records [12]. Our findings provide pragmatic context for that implementation vision: when eGFR and especially UACR are not measured consistently over time, opportunities to apply risk prediction tools, update risk estimates longitudinally and classify CKD risk in a timely manner may be missed [12,13]. In this sense, the observed screening patterns are not simply process metrics; they are central to the feasibility of operationalizing biomarker-driven cardiorenal risk stratification in real-world T2D care.

Our sensitivity analyses restricted to individuals with both eGFR and UACR available at baseline yielded outcome patterns consistent with the primary analyses, supporting the overall findings to baseline biomarker completeness. Additionally, among CKD-free individuals with both biomarkers available at index, baseline UACR, eGFR, age, BMI, and insulin use were independently associated with the first occurrence of an abnormal eGFR or UACR measurement over 5 years (Table S6). These factors overlap conceptually with the routinely available predictors used in diabetes-specific 5-year CKD risk prediction models, further supporting the clinical relevance of complete biomarker capture for risk assessment [12].

Kidney events were the most frequent outcomes during follow-up of this cohort, including worsening UACR category, eGFR decline, and a smaller proportion progressing to end-stage kidney disease. This pattern is clinically relevant given prior evidence that CKD is common in a cohort of T2D managed across primary and endocrinology care settings [4] (approximately 40% prevalence). Against the established background of heightened cardiorenal vulnerability when kidney disease is present [8,9,10], our observed 5-year event rates reinforce the clinical importance of timely CKD detection and ongoing monitoring in people with T2D.

Heart failure was the most frequent cardiovascular event, aligning with the evolving concept of the cardio–renal–metabolic axis in T2D. Large observational studies suggest that HF may represent an early and predominant cardiovascular manifestation in type 2 diabetes, sometimes preceding coronary or cerebrovascular disease [28,29]. The high prevalence of CKD at baseline (36.2%) and the high proportion of individuals with a first occurrence of an abnormal eGFR or UACR measurement during follow-up (37%) may further amplify HF risk through mechanisms such as volume overload, neurohormonal activation, and accelerated vascular ageing [30]. This association was also reflected in our results, as in the cohort with CKD at baseline, cardiovascular events happened in 25.5% of patients, HF being the most frequent one (13.5%).

All-cause mortality in the overall cohort was 16.1% over 5 years and was substantially higher among individuals with CKD at baseline (27.8%). These findings are consistent with prior work, including Afkarian et al. [31] and other studies [32,33], which reported markedly higher long-term mortality among individuals with concomitant type 2 diabetes and CKD.

In addition, the use of reno-protective therapies, such as RAAS inhibitors and particularly SGLT2 inhibitors, was lower than expected, even among individuals with CKD, suggesting substantial room to improve the uptake of treatments proven to reduce clinical events in this very high-risk population.

Our study has several limitations but also important strengths. First, we relied on routinely collected secondary healthcare data, which may be affected by incomplete capture, coding errors, and residual confounding. Second, because part of the inclusion criteria incorporated prescriptions for glucose-lowering agents, some individuals without confirmed type 2 diabetes may have been included, particularly given the expanding use of these therapies for indications beyond glycemic control. Third, part of the study period overlapped with the COVID-19 pandemic, which may have disrupted routine outpatient care and laboratory testing—especially albuminuria assessment—and could have contributed to the low screening rates observed.

Fourth, because eGFR and UACR testing was not uniform, CKD ascertainment and CKD-related outcomes may be influenced by measurement frequency, particularly given that CKD during follow-up was defined using the first pathological eGFR or UACR value in routine data. This approach is consistent with other real-world studies conducted in similar settings [2,21,34], but it may have introduced misclassification and could overestimate CKD prevalence and cumulative proportions compared with strictly KDIGO-confirmed CKD. This choice reflects the pragmatic considerations of epidemiological research while recognizing the inherent limitations in disease classification. Accordingly, our results should be interpreted within the context of a broad CKD definition based on a single abnormal value. Our estimates may include transient abnormalities and may therefore overestimate the proportions and burden of confirmed CKD. Fifth, CKD prevalence was estimated in the full eligible population rather than being restricted to individuals with complete baseline kidney testing, to reduce selection bias; however, 16% lacked eGFR and 30% lacked UACR at baseline, meaning that some participants classified as not having CKD may have had undetected kidney damage, potentially underestimating the true burden. Notably, sensitivity analyses restricted to individuals with both biomarkers available yielded consistent findings. Sixth, outcomes were summarized as crude 5-year cumulative proportions rather than time-to-event analyses, without adjustment for censoring or death as a competing risk for non-fatal outcomes. These estimates reflect the observed event burden in the cohort and may not represent true 5-year risk. Readers should therefore interpret Figure 1 and Figure 2 as descriptive estimates of the observed event burden in this population rather than as adjusted actuarial 5-year risks. Finally, information on diabetes duration was not available; insulin use was used as a proxy for longer disease duration and/or greater clinical complexity, which may have resulted in residual confounding.

Notwithstanding these limitations, this study’s strengths include its large, contemporary nationwide sample, linkage across care settings, inclusion of clinically meaningful cardiorenal outcomes, and the explicit evaluation of kidney biomarker monitoring practices alongside outcomes. By foregrounding ascertainment, the analyses provide actionable evidence that substantial cardiorenal event burden in cross-settings-managed T2D occurs alongside gaps in the routine capture of the biomarkers required for CKD detection and prognostic classification. In this context, the findings are aligned with multidisciplinary consensus efforts, which highlight the importance of coordinated diabetes management across specialties and emphasize key areas such as cardiovascular risk control and diabetic kidney disease, supported by agreed-upon recommendations from diverse healthcare settings to improve patient management and outcomes [35]. Santamaria et al. [36] assessed the burden of CKD in a Spanish real-world database using a CKD definition based on a single eGFR and UACR measurement, consistent with the operational approach adopted in our study. To address the potential impact of CKD misclassification, they additionally conducted a sensitivity analysis requiring two consecutive eGFR measurements and obtained similar results. These findings may support the validity of this pragmatic approach as an alternative, and could reinforce the association between worsening kidney function, increasing albuminuria, and a higher risk of cardiovascular and kidney complications in clinical practice.

## 5. Conclusions

Among adults with T2D managed by endocrinologists, cardiorenal events, hospitalizations, and mortality were frequent over 5 years, although kidney event estimates should be interpreted in the context of the broad operational CKD definition used in this study as the first occurrence of an abnormal eGFR or UACR measurement. Regular measurements of eGFR and particularly UACR occurred in a minority of patients. Improving the consistency of kidney biomarker monitoring may facilitate earlier recognition and risk classification of CKD in routine care, aligning clinical practice with the biomarker-dependent risk stratification paradigms supported by existing evidence.

## Figures and Tables

**Table 1:** Demographic and clinical characteristics of the study population at baseline.

	Overall	No CKD at Baseline	With CKD at Baseline
N (%)	13,600 (100%)	8675 (63.8%)	4925 (36.2%)
**DEMOGRAPHIC VARIABLES**			
Age (years), mean (SD)	64.7 (12.8)	62.1 (12.7)	69.5 (11.6)
Sex (female), *n* (%)	6787 (49.9)	4574 (52.7)	2213 (44.9)
**ANTHROPOMETRIC MEASURES**			
BMI (kg/m^2^), mean (SD)	31.8 (6.5)	31.8 (6.6)	31.9 (6.4)
Distribution, *n* (%)			
<25	1011 (7.4)	634 (7.3)	377 (7.7)
25–<30 (overweight)	2609 (19.2)	1643 (18.9)	966 (19.6)
≥30 (obesity)	4606 (33.9)	2849 (32.8)	1757 (35.7)
Missing/not available	5374 (39.5)	3549 (40.9)	1825 (37.1)
**CLINICAL AND LABORATORY VARIABLES,** Mean (SD)			
Systolic blood pressure (mmHg)	134.9 (18.5)	134 (17.9)	136.6 (19.3)
Diastolic blood pressure (mmHg)	77.1 (11.2)	78.3 (10.8)	75.1 (11.7)
eGFR (mL/min/1.73 m^2^)	79.8 (22.6)	89.9 (13.8)	63 (24.3)
Missing/not available, *n* (%)	2144 (15.8)	1535 (17.7)	609 (12.4)
UACR (mg/g) Median (IQR)	12.7 (7.1–36.6)	8.6 (5.8–13.6)	49.8 (18.3–142.9)
Missing/not available, *n* (%)	4056 (29.8)	3070 (35.4)	986 (20)
HDL (mg/dL)	44.5 (12)	45.8 (12)	42.5 (11.6)
LDL (mg/dL)	100.4 (33.2)	103.7 (32.7)	95 (33.3)
Triglycerides (mg/dL)	169.9 (125.6)	160.2 (117.3)	186 (136.8)
HbA1c (%)	7.4 (1.6)	7.2 (1.5)	7.6 (1.6)
**COMORBIDITIES,** *n* (%)			
CKD	4925 (36.2)	0 (0)	4925 (100)
Peripheral artery disease	1197 (8.8)	549 (6.3)	648 (13.2)
Stroke	834 (6.1)	419 (4.8)	415 (8.4)
Myocardial infarction	661 (4.9)	352 (4.1)	309 (6.3)
Angina pectoris	1018 (7.5)	538 (6.2)	480 (9.7)
Heart failure	1215 (8.9)	468 (5.4)	747 (15.2)
Atrial fibrillation/flutter	1161 (8.5)	493 (5.7)	668 (13.6)
Hypertension	7880 (57.9)	4420 (51)	3460 (70.3)
Hyperlipidemia	7664 (56.4)	4643 (53.5)	3021 (61.3)
Diabetic retinopathy	1125 (8.3)	572 (6.6)	553 (11.2)
Diabetic neuropathy	669 (4.9)	316 (3.6)	353 (7.2)
Obesity	4766 (35)	3060 (35.3)	1706 (34.6)
Hyperkalemia	380 (2.8)	136 (1.6)	244 (5)
**MEDICATIONS**, *n* (%)			
RAASi	8774 (64.5)	5004 (57.7)	3770 (76.5)
Beta blockers	3903 (28.7)	2011 (23.2)	1892 (38.4)
Calcium channel blockers	2913 (21.4)	1324 (15.3)	1589 (32.3)
Diuretics	3309 (24.3)	1387 (16)	1922 (39)
Lipid-lowering drugs	9351 (68.8)	5597 (64.5)	3754 (76.2)
Anti-platelet agents	5272 (38.8)	2793 (32.2)	2479 (50.3)
Glucose-lowering therapies (all)	12,027 (88.4)	7485 (86.3)	4542 (92.2)
Metformin	9153 (67.3)	6227 (71.8)	2926 (59.4)
SGLT-2 inhibitors	2493 (18.3)	1741 (20.1)	752 (15.3)
DPP-4 inhibitors	5403 (39.7)	3048 (35.1)	2355 (47.8)
GLP-1 RA	1336 (9.8)	887 (10.2)	449 (9.1)
Sulfonylurea	1999 (14.7)	1331 (15.3)	668 (13.6)
Meglitinides	833 (6.1)	332 (3.8)	501 (10.2)
Insulins	5929 (43.6)	3258 (37.6)	2671 (54.2)
Potassium binders (resins)	80 (0.6)	13 (0.1)	67 (1.4)

**Table 2:** Long-term clinical outcomes. Cumulative proportion of first kidney, cardiovascular, hospitalization, and all-cause mortality events over a 5-year observation period in the overall population and stratified by baseline chronic kidney disease (CKD) status.

Clinical Outcomes	Overall	No CKD at Baseline	With CKD at Baseline
N	13,600	8675	4925
Kidney events, *n* (%)	4226 (31.1)	1992 (23)	2234 (45.4)
Decline of eGFR ≥ 30%, *n* (%)	2318 (17)	1050 (12.1)	1268 (25.7)
Worsening of UACR category *, *n* (%)	2607 (27.3)	1199 (21.4)	1408 (35.7)
ESKD, *n* (%)	159 (1.2)	0 (0)	159 (3.2)
Cardiovascular events, *n* (%)	2532 (18.6)	1274 (14.7)	1258 (25.5)
Myocardial infarction	363 (2.7)	192 (2.2)	171 (3.5)
Stroke	560 (4.1)	287 (3.3)	273 (5.5)
Heart failure	1201 (8.8)	535 (6.2)	666 (13.5)
Peripheral artery disease	710 (5.2)	388 (4.5)	322 (6.5)
Hospitalizations ‡ (all cause), *n* (%)	3393 (24.9)	1862 (21.5)	1531 (31.1)
Hospitalizations ‡ due to HF, *n* (%)	380 (2.8)	152 (1.8)	228 (4.6)
Mortality (all cause), *n* (%)	2185 (16.1)	815 (9.4)	1370 (27.8)

* Worsening of UACR calculated over the total number of patients with available UACR value. ‡ Hospitalizations were defined as admissions lasting more than 24 h.

**Table 3:** Chronic kidney disease (CKD) screening practices during the 5-year follow-up in the overall population and according to baseline CKD status.

CKD Screening	Overall	No CKD at Baseline	With CKD at Baseline
N	13,600	8675	4925
Patients with at least 1 annual eGFR measurement, *n* (%)	5316 (39.1)	3696 (42.6)	1620 (32.9)
Average number of eGFR measurements in 5 years	2.9 (2.4)	3 (2.3)	2.7 (2.5)
Patients with at least 1 annual UACR measurement, *n* (%)	2260 (16.6)	1486 (17.1)	774 (15.7)
Average number of UACR measurements in 5 years	1.5 (1.6)	1.5 (1.6)	1.4 (1.7)
Average number of visits to Endocrinology in 5 years	4.5 (7.6)	4.6 (7.6)	4.4 (7.7)

## Data Availability

The data that support the findings of this study are available from Telómera^®^ but restrictions apply to the availability of these data, which were used under license for the current study, and so are not publicly available. Data are however available from the authors upon reasonable request and with permission of Telómera^®^.

## Supplementary Materials

The following supporting information can be downloaded at https://www.mdpi.com/article/10.3390/medsci14040506/s1, Figure S1: Study population flowchart; Table S1: Inclusion and exclusion criteria; Table S2: ICD-9 CM and ICD-10 CM diagnostic codes; Table S3: Anatomical Therapeutic Chemical classification codes (ATC); Table S4: Sensitivity analysis of long-term clinical outcomes. Cumulative proportion of first clinical events during a 5-year follow-up in individuals with available baseline eGFR and UACR measurements.; Table S5: Demographic and clinical characteristics of the study population used for the multivariate logistic regression model to identify baseline risk factors associated with the first occurrence of an abnormal eGFR or UACR measurement during a 5-year observation period.; Table S6: Baseline factors associated with the first occurrence of an abnormal eGFR or UACR measurement during a 5-year follow-up. Multivariate logistic regression analysis.

## Abbreviations

The following abbreviations are used in this manuscript:

ASCVD	Atherosclerotic cardiovascular disease
ATC	Anatomical Therapeutic Chemical
BMI	Body mass index
CKD	Chronic kidney disease
CVD	Cardiovascular disease
DPP4i	Dipeptidyl peptidase-4 inhibitors
eGFR	Estimated glomerular filtration rate
ESKD	End-stage kidney disease
GLP-1 RAs	Glucagon-like peptide-1 receptor agonists
HbA1c	Glycated hemoglobin
HDL	High-density lipoprotein
HF	Heart failure
ICD-9-CM	International Classification of Diseases, 9th Edition, Clinical Modification
ICD-10-CM	International Classification of Diseases, 10th Revision, Clinical Modification
IQR	Interquartile ranges
LDL	Low-density lipoprotein
MI	Myocardial infarction
PAD	Peripheral artery disease
RAASi	Renin–angiotensin–aldosterone system inhibitors
SD	Standard deviations
SGLT2i	Sodium-glucose cotransporter-2 inhibitors
UACR	Urine albumin-to-creatinine ratio
