# Supplementary material for: Five-Year Cardiorenal Outcomes and Longitudinal Chronic Kidney Disease Screening in People with Type 2 Diabetes Managed by Endocrinologists: A Nationwide Retrospective Cohort Study
Source: Med Sci (Basel). 2026 Aug 21;14(4):506. doi: 10.3390/medsci14040506 (PMC13515383; doi:10.3390/medsci14040506)
Supplement: Supplementary file 1 [file medsci-14-00506-s001.zip › Additional file 1_Suplementary Tables.pdf]

Five-Year Cardiorenal Outcomes and Longitudinal Chronic Kidney Disease Screening in People with Type 2 Diabetes Managed by Endocrinologists: A Nationwide Retrospective Cohort Study

Supplementary Tables

Table S1. Inclusion and exclusion criteria
Table S2. ICD-9 CM and ICD-10 CM diagnostic codes
Table S3. Anatomical Therapeutic Chemical classification codes (ATC)
Table S4. Sensitivity analysis of long-term clinical outcomes
Table S5. Demographic and clinical characteristics of the study population used for the multivariate logistic regression model
Table S6. Baseline factors associated with the first occurrence of an abnormal eGFR or UACR measurement during a 5-year follow-up

Table S1. Inclusion and exclusion criteria.

Inclusion criteria	Exclusion criteria
a) Age ≥ 18<br>b) Presence of diagnosis code for T2D, or at least one HbA1c value ≥6,5% or prescription fill for any antidiabetic drug<br>c) At least 1 visit to a national Endocrinology service at the time of or after T2D diagnosis, first recording of HbA1c value ≥6.5% or first prescription fill of any antidiabetic drug<br>d) Patients who have a minimum of 1 year look back recorded data before index date 30 November 2023<br>e) Patients with a minimum of ≥2 health records in the database (to ensure regular follow-up)<br>f) To be in the prescription program for obtaining medical prescriptions (with a proven record of the daily dosage, time interval and duration of each treatment administered, with at least 2 prescriptions during the follow-up period)	a) All patients with T1D, defined using the following assumptions and codes, as appropriate:<br>- Presence of diagnosis code for type 1 diabetes (T1D) **or**<br>- Patients <30 years of age with insulin as first diabetes medication or<br>- Patients with only insulin (either short-, long-acting or both) as the only diabetes medication<br>b) All patients with gestational diabetes (identified by the corresponding diagnosis code)<br>c) Patients transferred to other centers, displaced or out-of-area patients.<br>d) Permanently institutionalized patients

T2D: type 2 diabetes; HbA1c: glycated hemoglobin; T1D: type 1 diabetes.

Table S2. ICD-9 CM and ICD-10 CM diagnostic codes.

Disease	ICD-10 code	ICD-9 code

Type 2 Diabetes	E11	250.x1; 250.x3
Chronic Kidney Disease (CKD)	N17-N19, I12, I13, N08.3, E10.2, E11.2, E12.2, E13.2, E14.2, Z49, Z99.2	584-586, 250.4, 581.81, 583.81, 403-404, V45.1, V56, 996.56
CKD - Diabetic	E10.2, E11.2, E12.2, E13.2, E14.2, N08.3	250.4, 581.81, 583.81
CKD - Hypertensive	I12, I13	403-404
CKD - Dialysis	Z99.2, Z49, T85.69	V45.1, V56, 996.56
Peripheral artery disease (PAD)	I70.2, I73.9, I74.2-9	440.2, 443.9, 444.2-9
Stroke	I60-I63	430-432, 433.X1, 434.X1
Myocardial infarction (MI)	I21, I22, I25.2	410, 412
Angina pectoris	I20	411, 413
Heart failure (HF)	I50, I11.0, I13.0, I13.2	428, 402.01, 402.11, 402.91, 404.01, 404.11, 404.13, 404.91,404.93
Atrial fibrillation/flutter	I48	427.3, 427.4
Hypertension	I10-I15	401-405
Hyperlipidemia	E78	272
Diabetic retinopathy	E10.31-E10.35, E11.31-E11.35, E13.31-E13.35	250.5, 362.0
Diabetic neuropathy	E10.40-E10.43, E11.40-E11.43, E13.40-E13.43	250.6, 337.1, 357.2,
Obesity	E66.0, E66.1, E66.2, E66.8, E66.9	278.0
Hyperkalemia	E87.5	276.7

ICD-9 CM: International Classification of Diseases 9th Edition Clinical Modification; ICD-10 CM: International Classification of Diseases, 10th Revision, Clinical Modification.

Table S3. Anatomical Therapeutic Chemical classification codes (ATC).

Medication	ATC code
RASi	C09A, C09B, C09C, C09D (excluding C09DX04), C03DA, C09XA
Beta blockers	C07
Calcium channel blockers	C08
Diuretics	C03A, C03C, C03D, C03E
Lipid-lowering drugs	C10
Anti-platelet agents	B01AC
Glucose-lowering therapies	
Metformin	A10BA02, or A10BD (in combination)
SGLT-2 inhibitors	A10BK01, A10BK02, A10BK03, A10BK04 or A10BD15, A10BD16, A10BD20, A10BD23 (in combination)
DPP-4 inhibitors	A10BH, or A10BD07, A10BD08, A10BD09, A10BD10, A10BD11, A10BD12, A10BD13 (in combination)
GLP-1 RAs	A10BJ
Sulfonylureas	A10BB
Metiglinides	A10BX02, A10BX03 or A10BD03, A10BD04, A10BD05 (in combination)
Insulins	A10AB, A10AC, A10AD, A10AE
Potassium binders	
New K+ binders	V03AE09, V03AE10
Resins	V03AE01

RASi: Renin–Angiotensin System inhibitors; SGLT-2: sodium-glucose cotransporter type2; DPP-4: dipeptidyl peptidase 4; GLP-1 RAs: glucagon-like peptide 1 receptor agonists; K+: Potassium.

Table S4. Sensitivity analysis of long-term clinical outcomes. Cumulative proportion of first clinical events during a 5-year follow-up in individuals with available baseline eGFR and UACR measurements.

Clinical outcomes	Overall	No CKD at baseline	With CKD at baseline
N (%)	9492 (100%)	5570 (58.7%)	3922 (41.3%)
**Kidney events,** n (%)	3292 (34.7)	1306 (23.4)	1986 (50.6)
Decline of eGFR ≥30%	1715 (18.1)	613 (11)	1102 (28.1)
Worsening of UACR category*	2216 (23.3)	867 (15.6)	1349 (34.4)
ESKD	79 (0.8)	0 (0)	79 (2)
**Cardiovascular events**, n (%)	1696 (17.9)	719 (12.9)	977 (24.9)
Myocardial infarction	253 (2.7)	110 (2)	143 (3.6)
Stroke	394 (4.2)	173 (3.1)	221 (5.6)
HF	779 (8.2)	269 (4.8)	510 (13)
PAD	481 (5.1)	228 (4.1)	253 (6.5)
**Death and Hospitalizations**			
All-cause death, n (%)	1475 (15.5)	453 (8.1)	1022 (26.1)
Hospitalizations‡ (all), n (%)	2412 (25.4)	1176 (21.1)	1236 (31.5)
Hospitalization‡ due to heart failure, n (%)	226 (2.4)	49 (0.9)	177 (4.5)

*Worsening of UACR calculated over the total number of patients with available UACR value.

‡ Hospitalizations were defined as admissions lasting more than 24 hours.

CKD: Chronic kidney disease *per* study criteria; eGFR: estimated glomerular filtration rate; ESKD: end-stage kidney disease;.UACR: Urine albumin-to-creatinine ratio.

Table S5. Demographic and clinical characteristics of the study population used for the multivariate logistic regression model to identify baseline risk factors associated with the first occurrence of an abnormal eGFR or UACR measurement during a 5-year observation period.

Population CKD-free at baseline*	Overall	CKD-free at the end of the 5-year period	With abnormal eGFR or UACR at the end of the 5-year period
N (%)	5570 (100%)	4093 (73.5%)	1477 (26.5%)
**DEMOGRAPHIC VARIABLES**			
Age (years), mean (SD)	61.7 (12.1)	59.8 (12)	67.1 (10.7)
Gender (female), n (%)	2912 (52.3)	2122 (51.8)	790 (53.5)
**ANTHROPOMETRIC MEASURES**			
BMI (kg/m^2), mean (SD)	31.5 (6.6)	31.6 (6.8)	31.5 (5.9)
Missing/not available	2034 (36.5)	1538 (37.6)	496 (33.6)

**CLINICAL AND LABORATORY VARIABLES,** Mean (SD)			
Systolic blood pressure (mmHg)	133.5 (17.3)	132.8 (17.4)	135.3 (16.8)
Diastolic blood pressure (mmHg)	78.5 (10.7)	79 (10.6)	77.2 (11.1)
eGFR (mL/min/1.73 m^2)	89 (13.3)	91.8 (12.2)	81.5 (13.2)
Missing/not available	0	0	0
UACR (mg/g) Median (IQR)	8.6 (5.8-13.6)	8 (5.4-12.3)	10.7 (7-17.2)
Missing/not available	0	0	0
HDL (mg/dL)	45.9 (12.1)	46.1 (12.1)	45.5 (11.9)
LDL (mg/dL)	102.4 (32.1)	104.1 (32.5)	97.5 (30.6)
Triglycerides (mg/dL)	159 (114.7)	157.1 (115.1)	164.3 (113.5)
HbA1c (%)	7.2 (1.5)	7.2 (1.5)	7.3 (1.4)
**COMORBIDITIES,** n (%)			
Peripheral artery disease	316 (5.7)	205 (5)	111 (7.5)
Stroke	260 (4.7)	158 (3.9)	102 (6.9)
Myocardial infarction	206 (3.7)	138 (3.4)	68 (4.6)
Angina pectoris	347 (6.2)	219 (5.4)	128 (8.7)
Heart failure	246 (4.4)	142 (3.5)	104 (7)
Atrial fibrillation/flutter	257 (4.6)	146 (3.6)	111 (7.5)
Hypertension	2930 (52.6)	2038 (49.8)	892 (60.4)
Hyperlipidemia	3081 (55.3)	2221 (54.3)	860 (58.2)
Diabetic retinopathy	351 (6.3)	229 (5.6)	122 (8.3)
Diabetic neuropathy	204 (3.7)	132 (3.2)	72 (4.9)
Obesity	2057 (36.9)	1487 (36.3)	570 (38.6)
Hyperkalemia	76 (1.4)	47 (1.1)	29 (2)
**MEDICATIONS**, n (%)			
RAASi	3209 (57.6)	2175 (53.1)	1034 (70)
Beta blockers	1223 (22)	779 (19)	444 (30.1)
Calcium channel blockers	819 (14.7)	489 (11.9)	330 (22.3)
Diuretics	738 (13.2)	461 (11.3)	277 (18.8)
Lipid-lowering drugs	3655 (65.6)	2551 (62.3)	1104 (74.7)
Anti-platelet agents	1776 (31.9)	1190 (29.1)	586 (39.7)
Glucose-lowering therapies (all)	4890 (87.8)	3541 (86.5)	1349 (91.3)
Metformin	4103 (73.7)	2968 (72.5)	1135 (76.8)
SGLT-2 inhibitors	1185 (21.3)	864 (21.1)	321 (21.7)
DPP-4 inhibitors	1968 (35.3)	1380 (33.7)	588 (39.8)
GLP-1 RA	578 (10.4)	442 (10.8)	136 (9.2)
Sulfonylureas	899 (16.1)	624 (15.2)	275 (18.6)
Meglitinides	183 (3.3)	119 (2.9)	64 (4.3)
Insulins	2060 (37)	1451 (35.5)	609 (41.2)
Potassium binders (resins)	6 (0.1)	5 (0.1)	1 (0.1)

*All individuals had both eGFR and UACR measurements and did not present CKD at baseline. BMI: body mass index; CKD: chronic kidney disease per study criteria; DPP-4: dipeptidyl peptidase 4; eGFR: estimated glomerular filtration rate; GLP-1 RA: glucagon-like peptide 1 receptor agonists; HbA1c: glycated hemoglobin; HDL: high-density lipoprotein; LDL: low-density lipoprotein; RAASi: renin–angiotensin–aldosterone system inhibitors; SGLT-2: sodium-glucose cotransporter type 2; UACR: Urine albumin-to-creatinine ratio.

Table S6. Baseline factors associated with the first occurrence of an abnormal eGFR or UACR measurement during a 5-year follow-up. Multivariate logistic regression analysis.

Variable	OR	95% CI	p-value
eGFR	0.940	0.932-0.948	<0.001
UACR	1.070	1.057-1.084	<0.001
BMI	1.023	1.009-1.036	<0.001
Age	1.015	1.005-1.025	0.0022
Use of insulins	1.278	1.083-1.509	0.0037
SBP	1.005	1.000-1.010	0.0574

Model fit indices:.

AIC: Akaike information criterion = 3530.914.

BIC: Bayesian information criterion= 3574.034.

R^2 Nagelkerke= 0.236.

BMI: body mass index; eGFR: estimated glomerular filtration rate; CI: confidence interval; OR: odds ratio; SBP: systolic blood pressure; UACR: urine albumin-to-creatinine ratio.
