# Supplementary material for: Five-Year Cardiorenal Outcomes and Longitudinal Chronic Kidney Disease Screening in People with Type 2 Diabetes Managed by Endocrinologists: A Nationwide Retrospective Cohort Study
Source: Med Sci (Basel). 2026 Aug 21;14(4):506. doi: 10.3390/medsci14040506 (PMC13515383; doi:10.3390/medsci14040506)
Supplement: Supplementary file 1 [file medsci-14-00506-s001.zip › Additional file 2_Suplementary Figure.pdf]

Five-Year Cardiorenal Outcomes and Longitudinal Chronic Kidney Disease Screening in People with Type 2 Diabetes Managed by Endocrinologists: A Nationwide Retrospective Cohort Study

Figure S1. Study population flowchart.

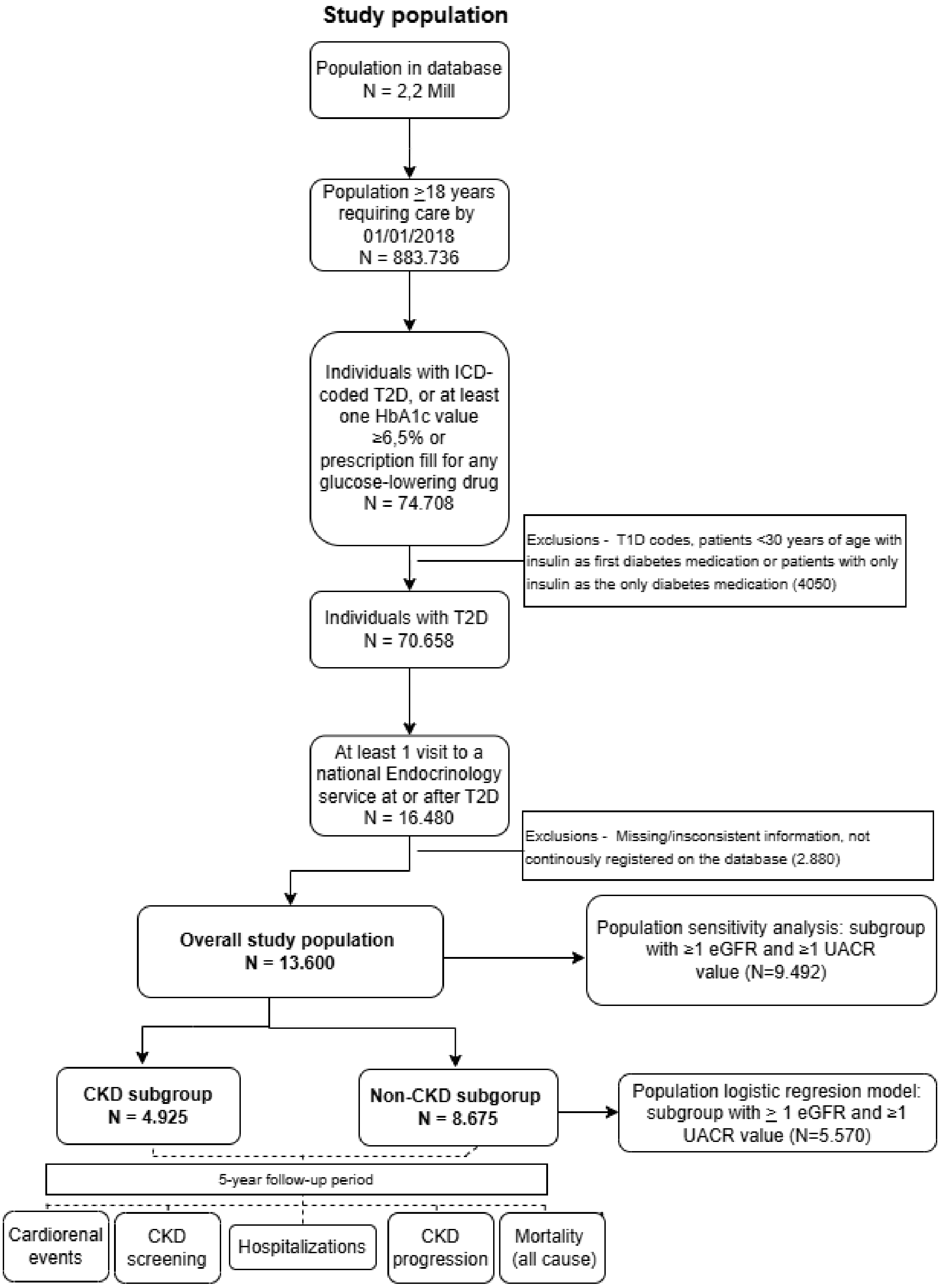

T2D: type 2 diabetes; ICD: International Classification of Diseases; HbA1c: glycated hemoglobin; T1D: type 1 diabetes; eGFR: estimated glomerular filtration rate; UACR: Urine albumin-to-creatinine ratio; CKD: chronic kidney disease
